# YTHDF2 enhances proliferation and metastasis of nasopharyngeal carcinoma by mediating m6A modification in destabilizing FOXO1 mRNA

**DOI:** 10.1080/15384047.2025.2582349

**Published:** 2025-12-10

**Authors:** Peng Yu, Wenyang Wei, Xinyun Peng, Jiemei Ye, Yuanli Ji, Bin Zhang, Yonglin Cai

**Affiliations:** a School of Clinical Medicine, Guilin Medical University, Guilin, Guangxi, People's Republic of China; b Department of Medical Laboratory, Liuzhou Worker's Hospital, Liuzhou, Guangxi, People's Republic of China; c Medical Laboratory, Wuzhou Red Cross Hospital, Wuzhou, Guangxi, People's Republic of China; d Guangxi Health Commission Key Laboratory of Molecular Epidemiology of Nasopharyngeal Carcinoma, Wuzhou Red Cross Hospital, Wuzhou, Guangxi, People's Republic of China; e Department of Radiation Oncology, Wuzhou Red Cross Hospital, Wuzhou, Guangxi, People's Republic of China

**Keywords:** Nasopharyngeal carcinoma, m6A, methylation modification, YTHDF2, FOXO1

## Abstract

**Background:**

Aberrant N6-methyladenosine (m6A) modification is linked to cancer development and progression. However, the role of YTH N6-methyladenosine RNA binding protein F2 (YTHDF2), an m6A ‘reader protein’, in nasopharyngeal carcinoma (NPC) is poorly understood. This study aimed to clarify the role and mechanism of YTHDF2 in NPC development.

**Methods:**

Bioinformatics analysis was performed to identify the differential expression, prognostic value, and enriched pathways of YTHDF2 in patients with NPC. Quantitative PCR, western blotting, and immunohistochemistry were used to detect mRNA and protein expression. Biological function of YTHDF2 was investigated using *in vitro* experiments, including proliferation, wound healing, and invasion assays. RNA immunoprecipitation sequencing (RIP-seq), RIP-qPCR, and Methylated RNA immunoprecipitation sequencing (MeRIP-seq) were employed to determine if YTHDF2 modulates forkhead box O1 (FOXO1) expression through m6A modification.

**Results:**

YTHDF2 mRNA and protein levels were significantly increased in the NPC tissues and cell lines. Higher expression of YTHDF2 was associated with a poorer prognosis. Overexpressing YTHDF2 enhanced the NPC cell proliferation, migration, and invasion. Conversely, YTHDF2 knockdown inhibited these phenomena. Gene set enrichment analysis revealed that FOXO1-related signaling pathways were enriched in the YTHDF2-activated group. Mechanistically, YTHDF2 overexpression inhibited FOXO1 expression in NPC cells. RIP-seq, RIP-qPCR, and MeRIP-seq assays confirmed that YTHDF2 was bound to FOXO1 mRNA, reducing its stability and accelerated degradation.

**Conclusion:**

YTHDF2 potentially functions as an oncogene in NPC by binding to the m6A site of FOXO1, reducing its expression, thereby promoting malignant behavior. It may also be a viable biomarker and therapeutic target for NPC.

## Introduction

Nasopharyngeal carcinoma (NPC), a primary epithelial cell carcinoma originating from the nasopharyngeal mucosa, is undifferentiated and prevalent in endemic areas.^1^ This cancer has a global presence but is notably endemic among certain populations in East and Southeast Asia, North Africa, and particularly in the Guangdong and Guangxi provinces of China. Early diagnosis of NPC is challenging because the condition often goes unnoticed for long periods, leading to a high rate of misdiagnosis and missed opportunities for early intervention.^2^ Although radiotherapy has significantly advanced in NPC treatment, recurrence and metastasis remain significant challenges.^3^ The exact etiology of NPC is unclear, highlighting the urgent need to explore the mechanisms behind its growth and spread. It is crucial to develop effective therapeutic strategies, as this could greatly enhance the treatment efficacy and improve the prognosis of NPC patients.

N6-methyladenosine (m6A) methylation modification occurs at the sixth position *N* of adenosine monophosphate. Research has revealed that m6A is vital for the biological processes of tumor cells, including proliferation, invasion, and metastasis, and is crucial for tumor progression.^4^ FTO (Fat mass and obesity-associated protein), an m6A demethylase, removes m6A modifications from OTUB1 (OTU Deubiquitinase, Ubiquitin Aldehyde Binding 1) mRNA, which promotes the expression of OTUB1, inhibiting radiotherapy-induced ferroptosis and causing radiotherapy resistance in NPC.^5^ Wilms' tumor 1-associating protein (WTAP) -mediated m6A modification enhances the stability of the lncRNA DIAPH1-MS1, ultimately promoting NPC proliferation and metastasis.^6^ These findings underscore the importance of m6A modifications in NPC development.

YTHDF2 (YTH N6-Methyladenosine RNA Binding Protein F2), an m6A ‘reader protein’, has been demonstrated to have the ability to modulate mRNA stability.^7^ Moreover, it is involved in several biological processes such as cell proliferation, apoptosis, differentiation, and inflammation. Furthermore, YTHDF2 has been identified as a biomarker capable of predicting the prognosis of various cancers.^8^ Current research has revealed that YTHDF2 is upregulated in ovarian cancer tissues. Its expression is positively correlated with clinical staging and pathological grading in ovarian cancer.^9^ In hepatocellular carcinoma, an inverse relationship was observed between YTHDF2 expression and patient survival rates.^10^ In multiple myeloma, YTHDF2 is significantly overexpressed and identified as an independent prognostic factor that can predict the survival outcomes of patients with this disease.^11^ A recent study in radiation-induced lung injury demonstrated that METTL3-mediated m⁶A modification of FOXO1 mRNA promotes its YTHDF2-dependent degradation, thereby activating AKT/ERK signaling.^12^ To date, there is a lack of research on the post-transcriptional modification of YTHDF2 in NPC. Therefore, elucidation of the regulatory mechanisms of YTHDF2 is of critical importance as it may provide an important basis for the prevention and treatment of NPC.

This study examined YTHDF2 expression in NPC and its role in promoting malignant behavior of NPC cells *in vitro*. Additionally, our findings indicated that YTHDF2 negatively regulates the expression of forkhead box O1 (FOXO1) via m6A modification.

## Materials and methods

### Bioinformatics analysis of YTHDF2 in NPC gene expression omnibus (GEO) datasets

Bioinformatics analysis was conducted using R software (version 4.3.1). The GSE12452, GSE180272, GSE53819, GSE61218, and GSE64634 datasets downloaded from the GEO database were combined using the “sva” package, which included 89 NPC cases and 56 normal controls. The differential expression of genes between malignant and nonmalignant epithelial cells was analyzed using the single-cell NPC dataset, GSE150430. The association between YTHDF2 expression and progression-free survival (PFS) in NPC patients from GSE102349 was assessed using the ‘survminer’ and ‘survival’ packages.

### Gene set enrichment analysis (GSEA)

The GSE102349 dataset samples were split into high and low YTHDF2 expression groups based on the median value. Subsequently, the “clusterProfiler” R package was utilized to conduct and visualize the GSEA.

### Cells and clinical samples

NPC cell lines 5-8F, CNE1 and HONE1, along with the immortalized normal nasopharyngeal epithelial cell line NP69, were provided by Guangxi Medical University, China. NPC cells were maintained in DMEM high glucose medium with 10% fetal bovine serum and 1% penicillin-streptomycin at 37 °C in a 5% CO_2_ incubator. NP69 cells were cultured in a medium comprising an equal mixture of DK-SFM with growth factors and Epilife medium.

We collected 17 primary fresh NPC tissues, 15 rhinitis tissues, 45 paraffin-embedded NPC tissues, and 26 paraffin-embedded rhinitis tissues from the Wuzhou Red Cross Hospital, China. In this study, all patients with NPC underwent a comprehensive evaluation encompassing medical history, clinical manifestations, imaging studies, laboratory tests, and pathological examination results to establish the diagnosis and determine the disease stage. The pathological examination results were utilized as the definitive diagnostic criterion. The study cohort included only patients who were newly diagnosed and had not previously received any anti-tumor treatment. Patients with a history of other malignancies were excluded from the study. Detailed patient information is provided in the Supplementary Material 1. The Ethics Committee of Wuzhou Red Cross Hospital granted approval for this study (Approval No. LL2020−02). Participants signed an informed consent form.

### Main reagents

DMEM high-glucose medium was acquired from Gibco (America, C11995500BT), and FBS was acquired from Sigma (Australia, F8318). PCMV6-entry-YTHDF2 plasmid (China, Wuxi, RC200038) and empty vector plasmid (China, Wuxi, PS100001) were obtained from OriGene Technologies. FOXO1 antibody (America, 2880) was procured from CST, and YTHDF2 antibody (China, Wuhan, Polyclonal Antibody for WB, IHC, IP, 24744−1-AP) and GAPDH antibody (China, Wuhan, Monoclonal Antibody for WB, IHC, 60004−1-Ig) were bought from Proteintech. Lipofectemine−3000 Transfection (Lithuania, L3000075) and Lipofectemine RNAiMAX Transfection (Lithuania, 13778−150) were acquired from ThermoFisher.

### RT-qPCR (Reverse transcription-quantitative PCR)

The RNA Easy Fast Tissue/Cell Kit (TIANGEN, DP451) was used for the extraction of total RNA from tissues and cells. The cDNA synthesis was performed using DNase I, RNase-free (ThermoFisher, EN0529) and Reverse Transcription Kit (ThermoFisher, M1631). RT-qPCR was conducted using the SYBR Green method. The reaction procedure was as follows: pre denature at 95 °C for 2 minutes; Denaturation at 95 °C for 15 seconds, annealing and extension at 60 °C for 1 minute, a total of 40 cycles. GAPDH served as an internal reference for normalization. Target gene expression levels were calculated using the 2^−ΔΔCt^ algorithm. Supplementary Material 2 detailed the methodologies employed for RNA extraction and cDNA synthesis, as well as the primer sequences utilized in RT-qPCR assays.

### Western blotting

Total protein was extracted using a cell lysis buffer (Beyotime, P10013B) supplemented with a 1% protease inhibitor. Protein concentrations were detected using a BCA protein quantification kit. Samples were prepared by mixing with an equal volume of 4 × sample buffer (ThermoFisher, NP0007) and heating at 70 °C for 10 min to denature the proteins. Protein was electrophoresed on a precast gel (ThermoFisher, NP0301BOX) at 200 V for 30 min and transferred to a polyvinylidene fluoride membrane (Millipore, IPVH00010) at 250 mA for 1.5 h. The membrane was blocked with buffer for 15 min at room temperature and washed three times with 1 × TBST. A primary antibody was applied, and the membrane was incubated overnight at 4 °C. Following incubation, the membrane was washed three times with 1 × TBST and subsequently incubated at room temperature with a secondary antibody conjugated to horseradish peroxidase for 1.5 h with shaking. Protein bands were visualized with a chromogenic solution, and a gel imaging analyzer was used to scan the images. Protein band gray values were quantified with ImageJ v1.53t software.

### Immunohistochemical (IHC) staining

Paraffin-embedded tissue sections were placed in a 60 °C incubator for 3 h and deparaffinized with xylene. The sections were then rehydrated through a graded series of alcohol concentrations (100%–75%). Antigen retrieval was performed using a high-temperature–high-pressure method. Endogenous peroxidase activity was inhibited using 3% H_2_O_2_. Sections were incubated overnight at 4 °C with the primary antibody, then for 30 min at 37 °C with a horseradish peroxidase-conjugated secondary antibody. YTHDF2 expression was visualized using a diaminobenzidine substrate for color development, followed by counterstaining of the cell nuclei with hematoxylin. The sections were differentiated using hydrochloric acid-alcohol. Two independent pathologists assessed and scored the staining's intensity and distribution. The criteria for judging the results of immunohistochemical reactions are shown in table 4 of the article.^13^

### Cell transfection

Detailed cell transfection procedures and reagents are provided in Supplementary Material 3.

### Cell proliferation and colony formation assays

The impact of YTHDF2 on cell proliferation and colony formation was evaluated using the cell counting kit−8 (CCK−8) assay (Dojindo, CK04) and a colony formation assay. Post-transfected NPC cells were seeded into 96-well plates at 1000 cells per well for the cell proliferation assay. Five replicate wells were established for both the experimental and control groups. Cells were incubated with CCK−8 reagent at 37 °C for 1.5 h, and absorbance was detected at 450 nm every 24 h for 5 days. In the colony formation assay, transfected NPC cells were seeded at a density of 500 cells per well in six-well plates and incubated at 37 °C for approximately 14 days. The cells were fixed in 4% paraformaldehyde, stained using 0.1% crystal violet, and quantified via ImageJ v1.53t software.

### Wound healing and transwell invasion assays

For the wound healing assay, NPC cells were transiently transfected and seeded into a 12-well plate. After the cells reached approximately 90%−95% confluence (subconfluence), a sterile wound scratch was created across the monolayer using a pipette tip. Any detached cells and debris within the scratch were carefully washed away with phosphate-buffered saline (PBS). Each well was then replenished with serum-free medium. Wound closure was monitored and photographed under a microscope at specific time points (e.g., 0, 12, 24, 48 h). The rate of scratch healing was quantified by measuring the remaining wound area at each time point relative to the initial wound area (0 h) using ImageJ v1.53t software.

For the Transwell invasion assay, a matrigel layer was applied to the surface of the invasion chamber. In a Transwell plate, transfected NPC cells were plated into the upper chamber, which was then filled with serum-free medium. Serum-containing medium filled the lower chamber. Cotton swabs were used to gently remove non-invading cells on the upper surface of the membrane after 24 h of incubation. The chamber was washed, fixed in 4% paraformaldehyde, stained with 0.1% crystal violet, and examined under a microscope.

### RNA immunoprecipitation sequencing (RIP-Seq), RIP-qPCR, and methylated RNA immunoprecipitation sequencing (MeRIP-seq)

RIP-seq and RIP-qPCR experiments were performed by Wuhan Seqhealth Company, leveraging their expertise in RNA-protein interaction analysis. MeRIP-seq was performed by the Shanghai Genesky Company, which specializes in the enrichment and analysis of methylated RNA molecules. The experimental methods and validation data for RIP-seq, RIP-qPCR, and MeRIP-seq were detailed in Supplementary Material 4.

### Statistical analysis

R (version 4.3.1) and GraphPad Prism (version 8.0) software were applied to perform the statistical analyses. Each experimental condition was replicated at least thrice to ensure reliability and reproducibility. Comparison between two or more groups was performed using a two-tailed unpaired t-test or one-way ANOVA. As the data do not adhere to a normal distribution, we employed non-parametric methods for group comparisons. Specifically, the rank sum test was used: the Mann-Whitney U test for comparisons between two groups and the Kruskal-Wallis test for comparisons among more than two groups. Wilcoxon test was used to analyze the mRNA expression data from clinical tissue samples. Statistical significance was defined as two-tail *P*-value < 0.05.

## Results

### YTHDF2 was upregulated in NPC cells and associated with poor prognosis of NPC

**Figure 1.**
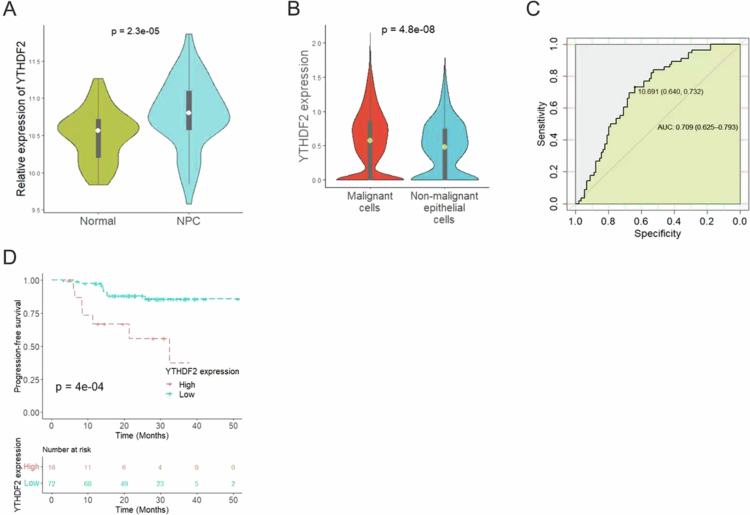
Analysis of the expression of YTHDF2 and its impact on the progression of NPC. (A) Comparison of the transcription levels of YTHDF2 in the combined GSE12452, GSE180272, GSE53819, GSE61218, and GSE64634 datasets. (B) Comparison of the expression level of YTHDF2 between malignant and nonmalignant cells in single-cell NPC dataset GSE150430. (C) ROC curve analysis of YTHDF2 in diagnosing NPC. (D) Kaplan–Meier survival curve for YTHDF2 expression of NPC in GSE102349.

Bioinformatics analysis revealed a significant upregulation of YTHDF2 transcript expression in NPC tissues (Figure 1A). Similarly, single-cell analysis of NPC showed significantly higher YTHDF2 expression in malignant cells compared to nonmalignant epithelial cells (Figure 1B). ROC curve analysis indicated that YTHDF2 exhibited moderate accuracy in diagnosing NPC, with a sensitivity of 64%, specificity of 73.2%, and an area under the curve (AUC) of 0.709 (Figure 1C). Kaplan–Meier survival curves demonstrated that patients with high YTHDF2 expression levels had significantly poorer PFS than those with low YTHDF2 expression levels (Figure 1D). The findings indicate that YTHDF2 could serve as a molecular marker for NPC diagnosis and prognosis.

**Figure 2.**
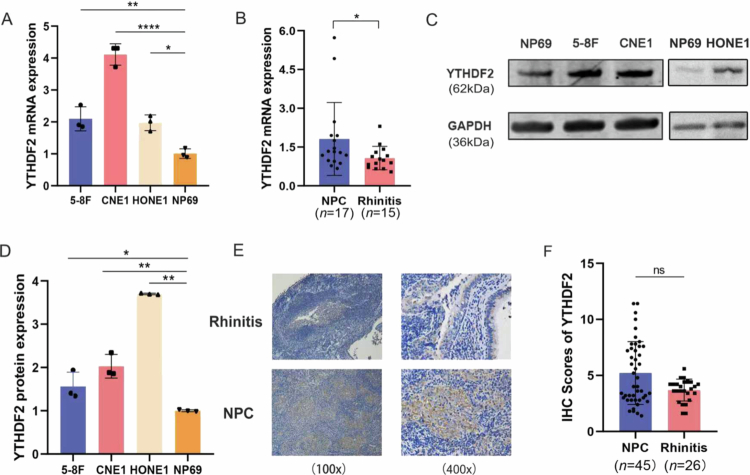
The expression of FOXO1 in the transcription and translation of NPC cells and tissues. (A) Comparison of the transcription level of YTHDF2 in NPC cell lines with NP69. (B) Comparison of the transcription level of YTHDF2 in NPC tissues with Rhinitis.(A: *n* = 3 biological replicates, one-way ANOVA, B: t-test). (C-D) Comparison of the protein level of YTHDF2 in NPC cell lines with NP69. (E-F) Comparison of the protein level of YTHDF2 in NPC tissues with Rhinitis. (D: *n* = 3 biological replicates, one-way ANOVA, F: t-test). *P < 0.05; **P < 0.01; ***P < 0.001; ****P < 0.0001; ns: not significant.

We subsequently analyzed YTHDF2 expression in both NPC cell lines and clinical tissues. YTHDF2 mRNA expression was significantly elevated in 5-8F, CNE1 and HONE1 cells as well as in NPC tissues compared to their respective controls (Figure 2A and B). Moreover, YTHDF2 protein expression was significantly elevated in 5-8F, CNE1 and HONE1 cells compared to NP69 cells (Figure 2C and D), whereas it was higher in NPC tissues compared to rhinitis tissues (Figure 2E and F) although the difference was statistically non-significant. YTHDF2 is upregulated in NPC and correlates with poor prognosis, identifying it as a putative oncogenic driver essential for NPC progression rather than a mere bystander.

### YTHDF2 promoted the proliferation, migration, and invasion of NPC cells *in vitro*

**Figure 3.**
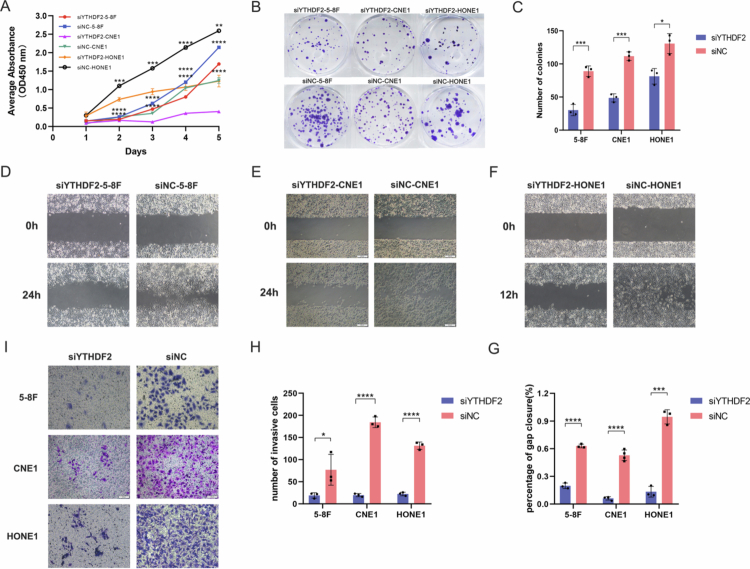
*In vitro* functional experiments of YTHDF2 knockdown in NPC cells (*n* = 3 biological replicates, student's t-test). (A-C) YTHDF2 knockdown inhibited the proliferation in 5-8F, CNE1 and HONE1cells. (D-G) YTHDF2 knockdown reduced the migration in 5-8F, CNE1 and HONE1 cells. (H-I) YTHDF2 knockdown reduced the invasive capabilities of 5-8F, CNE1 and HONE1 cells. *P < 0.05; **P < 0.01; ***P < 0.001; ****P < 0.0001.

**Figure 4.**
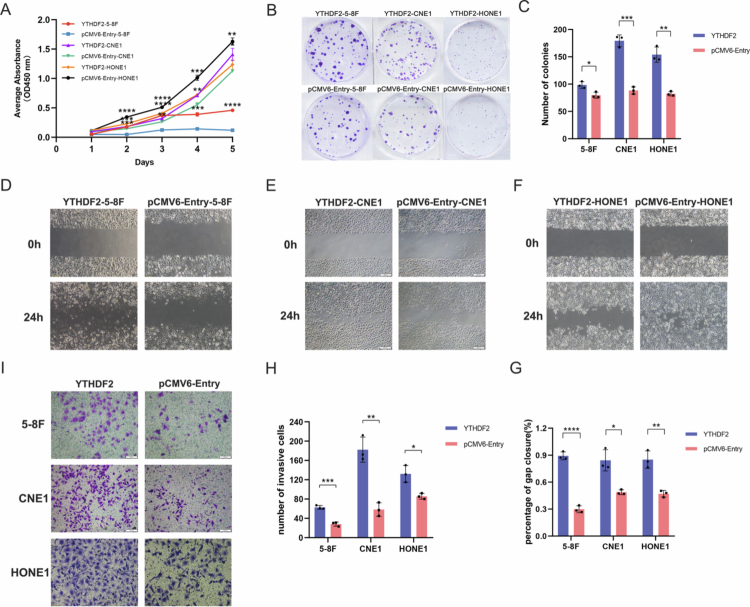
*In vitro* functional experiments of YTHDF2 overexpression in NPC cells (*n* = 3 biological replicates, student's t-test). (A-C) YTHDF2-transfected 5-8F, CNE1, and HONE1 cells exhibited accelerated proliferation. (D-G) Enhanced migratory capacity was observed in YTHDF2-overexpressing cells. (H-I) Matrigel invasion assays revealed significantly increased invasiveness following YTHDF2 overexpression. *P < 0.05; **P < 0.01; ***P < 0.001; ****P < 0.0001.

To elucidate YTHDF2's impact on the malignant behavior of NPC cells, we generated transient knockdown and overexpression constructs for YTHDF2 in the NPC cell lines 5-8F, CNE1 and HONE1. The CCK−8 assay revealed that si-YTHDF2 suppressed the proliferation of 5-8F, CNE1 and HONE1 cells (Figure 3A). The clone formation experiment showed that si-YTHDF2 decreased the number of colonies and reduced the cell proliferation capacity compared to the control cells (Figure 3B-C). A significant suppression of wound healing rates was observed in both YTHDF2 knockdown cell lines (Figure 3D–F), and a significant reduction in cell migration was detected in the Transwell assay (Figure 3G-H). Conversely, YTHDF2 overexpression enhanced the proliferation, migration, and invasive capabilities of 5-8F and CNE1 and HONE1 cells (Figure 4). The findings suggest that YTHDF2 promotes the cell malignant behavior of NPC *in vitro*.

### YTHDF2 inhibited FOXO1 expression by decreasing its mRNA stability in an m6A-dependent manner

**Figure 5.**
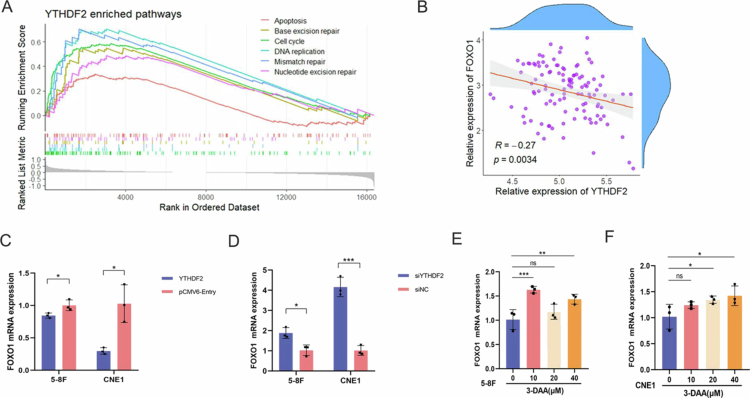
Correlation between YTHDF2 and FOXO1 expression (*n* = 3 biological replicates, C-D: t-test, E-F: one-way ANOVA). (A) GSEA in GSE102349 revealed enrichment pathways in the YTHDF2-activated group. (B) Correlation analysis between YTHDF2 and FOXO1 expression in GSE102349. Analysis of FOXO1 expression in 5-8F and CNE1 cells following YTHDF2 knockdown (C) or overexpression (D). Comparison of FOXO1 expression in NPC cell lines 5-8F (E) and CNE1 (F) which were treated with the m6A demethylase inhibitor 3-DAA for 48 h. *P < 0.05; **P < 0.01; ***P < 0.001; ns: not significant.

The involvement of m6A reader proteins, which identify m6A in RNA, is essential for determining the fate of N6-methylated RNA and for executing related biological functions.^14^ We performed GSEA to investigate the biological processes related to YTHDF2 expression. The results revealed that apoptosis, cell cycle, mismatch repair, DNA replication, nucleotide excision repair, and base excision repair were significantly enriched in the YTHDF2-activated group (Figure 5A). The FOXO family is vital for regulating cell cycle, apoptosis, autophagy, tumor suppression, and metabolism.^15^^,^^16^ FOXO1 has been widely reported to exert tumor-suppressive effects across diverse cancer types.^17-19^ Building on this evidence, we hypothesize that there may be some regulatory relationship between YTHDF2 and FOXO1, we focused on FOXO1 for our further assays. Further analysis identified a significant negative correlation between YTHDF2 and FOXO1 expression levels in GSE102439 (Figure 5B). Therefore, we speculated that YTHDF2 as m6A readers might be a regulator of FOXO1. To further explore the regulatory dynamics between YTHDF2 and FOXO1, we established the NPC cell lines 5-8F and CNE1 with either YTHDF2 deficiency or overexpression. We then quantified the FOXO1 expression within these cells. Our results indicated a pronounced increase in FOXO1 mRNA expression in NPC cells where YTHDF2 expression was suppressed (Figure 5C). Contrarily, YTHDF2 upregulation corresponded with a significant reduction in FOXO1 levels (Figure 5D), which is consistent with our bioinformatics analysis. These findings suggest a regulatory relationship between YTHDF2 and FOXO1.

The m6A demethylase inhibitor 3-Deoxyadenosine (3-DAA) was applied to both 5-8F and CNE1 cells and incubated for 48 h. Afterward, FOXO1 expression levels increased in both cell lines (Figure 5E-F). This finding suggests that FOXO1 mRNA in NPC cells may contain m6A modification sites.

**Figure 6.**
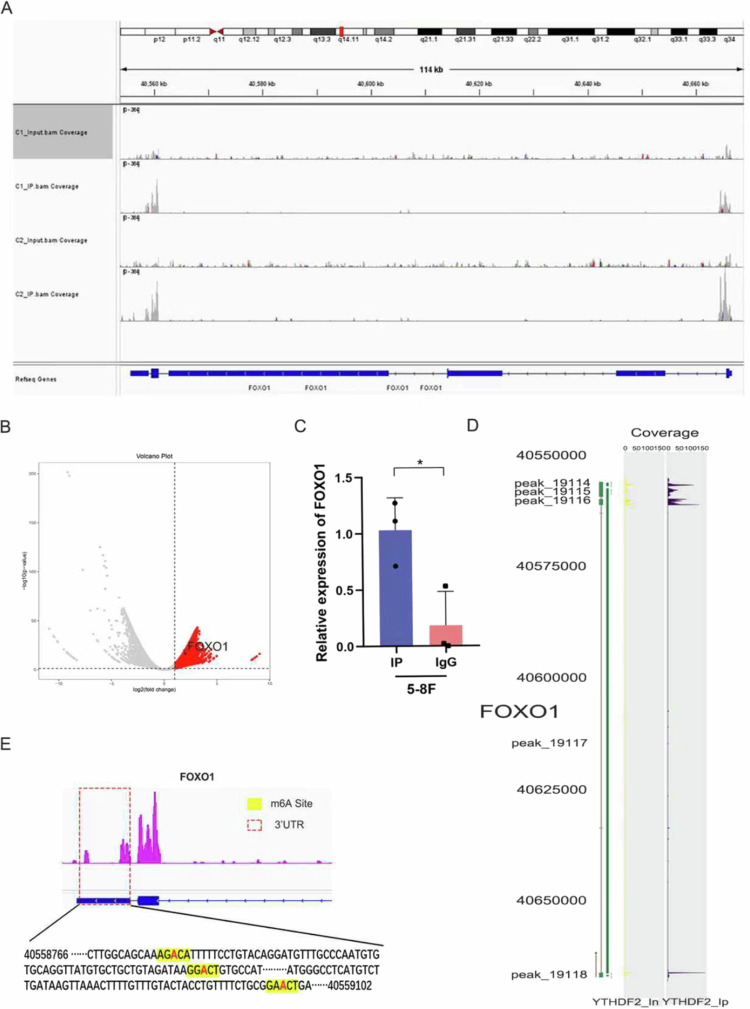
YTHDF2 participates in m6A-mediated regulation of FOXO1. (A) Distribution of m6A modification sites of FOXO1 mRNA in 5-8F. (B) Volcano plot of MeRIP-seq analysis for YTHDF2. (C) RIP-qPCR exhibited the relative expression of FOXO1. (D) YFHDF2 binding interval in FOXO1. (E) M6A modification site on the 3'UTR of FOXO1.

To understand the molecular mechanism by which YTHDF2 regulates FOXO1 expression, we performed MeRIP-seq in 5-8F cells (Figure 6A). MeRIP-seq analysis robustly validated the presence of m6A methylation sites in FOXO1 mRNA. Subsequent RIP-seq and RIP-qPCR experiments provided further evidence of the interaction between YTHDF2 and FOXO1 mRNA (Figure 6B-D). Using YTHDF2-specific antibodies, we immunoprecipitate the YTHDF2 protein together with its bound RNAs from cell lysates. The co-purified RNAs were then isolated from the complex and subjected to high-throughput sequencing. Figure 6B showed the volcano plot of YTHDF2 antibody (YTHDF2_IP) vs. negative control IgG (YTHDF2_In) (|log2FC| ≥ 1, FDR ≤ 0.05) in 5-8F cells, including FOXO1 (Figure 6B). Our results indicate that YTHDF2, functioning as an m6A reader, binds a wide array of target genes in addition to FOXO1, suggesting its extensive involvement in post-transcriptional regulation. Based on these findings, we performed RIP-qPCR to quantitatively verify FOXO1 binding to YTHDF2. In the 5-8F cell line, YTHDF2_IP significantly enriched FOXO1 mRNA compared with YTHDF2_In, indicating that YTHDF2 recognizes and binds to FOXO1 mRNA (Figure 6C). This result supports an interaction between YTHDF2 and FOXO1 mRNA. To enhance the reliability of the results, the binding sites of YTHDF2 protein on FOXO1 mRNA were mapped in 5-8F cells, elucidating that YTHDF2 can bind to and regulate the expression of FOXO1 in NPC cells (Figure 6D). The m6A site analyzed by Peak (Figure 6D) and MeRIP-seq overlaps (Figure 6A), confirming the canonical “reader”-mode binding of YTHDF2. We further identified the m6A modification site on the 3'UTR of FOXO1 (Figure 6E). Based on our findings, FOXO1 expression is significantly modulated by YTHDF2.

## Discussion

Aberrant m6A modifications are significantly linked to the development and progression of various malignant tumors. The YTH domain family proteins as m6A ‘readers’ directly bind and recognize m6A methylation in mRNA. YTHDF2 localizes to the m6A-modified mRNA in cytoplasm and promotes mRNA degradation. SUMOylation, a novel molecular mechanism for the functional regulation of YTHDF2, significantly enhances the binding affinity of YTHDF2 for m6A-modified mRNAs and crucially influences the regulation of post-transcriptional gene expression and tumorigenesis.^20^ O-GlcNAcylation could inhibit the YTHDF2 ubiquitination and increase its protein stability, which promotes the progression of HBV-associated hepatocellular carcinoma.^21^ Current research has demonstrated that YTHDF2 may act as both a tumor suppressor and an oncogene.^22^ YTHDF2 promotes the progression of gastric cancer,^23^ oral squamous cell carcinoma,^24^ hepatocellular carcinoma,^25^ prostate cancer,^26^ breast cancer^27^ and glioblastoma,^28^ but suppresses the development of colorectal cancer,^29^ osteosarcoma^30^ and melanoma.^31^ This diversity may be related to the extracellular microenvironment, tumor tissue heterogeneity, and associated upstream or downstream regulators. However, the role of YTHDF2 in NPC is still unclear. We utilized bioinformatics analysis, RT-qPCR, and IHC experiments to demonstrate upregulation of YTHDF2 mRNA and protein levels in NPC tissues and cell lines. Elevated YTHDF2 expression is associated with reduced Progression-Free Survival (PFS) in NPC patients. A series of cellular functional experiments confirmed that YTHDF2 promoted the proliferation, invasion, and migration abilities of NPC cells. These findings indicate that YTHDF2 exerts a tumor-promoting effect and its up-regulation may contribute to poor prognosis in NPC patients.

To explore the downstream pathways of YTHDF2 regulation, we performed GSEA and found that YTHDF2 regulates the cell cycle and apoptosis. FOXO1, a member of the forkhead transcription factor family, is crucial in regulating cancer cell proliferation, apoptosis, and migration, functioning as a key tumor suppressor in various cancers.^32-34^ Studies revealed that FOXO1 could suppress NPC growth, chemoresistance, and radioresistance. Zhao et al.^35^ reported that miR−3188 induced by FOXO1 inhibits cancer growth and enhances 5-fluorouracil sensitization in NPC by modulating the mTOR/PI3K/Akt/c-Jun axis. Li et al.^36^ demonstrated that FOXO1 suppresses tumor stemness and epithelial-mesenchymal transition while inducing cisplatin sensitivity in NPC cells by downregulating MYH9 expression through the PI3K/AKT/c-Myc/P53/miR-133a-3p axis. According to Deng et al.^37^, miR−613 inhibits the downstream STAT1/FOXO1 pathway in NPC cells, enhancing their radiosensitivity. STAT3 enhances NPC metastasis by modulating SRGN via the FoxO1-miR-148a-5p-CREB1 pathway.^38^ Chen et al.^39^ found that HOXB2 and FOXO1 synergistically regulate NPC progression and radioresistance. Studies have shown that METTL3-regulated m6A modification facilitates FOXO1 mRNA decay through YTHDF2 in radiation-induced lung injury and endometriosis-related infertility.^40^^,^^41^ Our further investigation indicated that YTHDF2 may mediate the m6A methylation modification of post-transcriptional FOXO1 and accelerate the degradation of FOXO1 mRNA, thereby promoting NPC progression. These findings would further enrich the m6A regulatory network in NPC, providing new insights and potential directions for improved diagnosis and treatment strategies.

However, some limitations exist in this study. First, we only performed functional experiments and mechanistic analyses in NPC cell lines. Second, the clinical value of the diagnostic and prognostic biomarkers of YTHDF2 was evaluated using retrospective data from the GEO dataset. Furthermore, *in vivo* experiments and prospective validation cohort studies will be required.

In conclusion, our study demonstrated that YTHDF2 promotes NPC cell proliferation, invasion, and migration by suppressing FOXO1 expression via m6A methylation. YTHDF2 could serve as a potential biomarker and therapeutic target for NPC.

## Supplementary Material

Supplementary Material Supplementary Material 4.

Supplementary Material Supplementary Material 3.

Supplementary Material Supplementary Material 2.

Supplementary Material Supplementary Material 1.

Supplementary Material Figure Legends.

## Data Availability

The data are available from the corresponding author upon reasonable request.
