# Supplementary material for: YTHDF2 enhances proliferation and metastasis of nasopharyngeal carcinoma by mediating m6A modification in destabilizing FOXO1 mRNA
Source: Cancer Biol Ther. 2025 Dec 10;26(1):2582349. doi: 10.1080/15384047.2025.2582349 (PMC12698064; doi:10.1080/15384047.2025.2582349)
Supplement: Supplementary Material — 4. [file KCBT_A_2582349_SM1420.docx]

Supplementary Material 4

RIP Experiment, Library Preparation, and Sequencing:

1. Cells were removed from the incubator and washed with 1× PBS. They were then digested with trypsin, followed by two washes with 1× PBS. The cells were centrifuged, and the supernatant was discarded.

2. The RIP lysis buffer was pipetted and gently blown into the cell mass to resuspend the cells. The suspension was placed on ice and lysed for 5 minutes, after which the lysate was retained for further processing.

3. The lysed samples were divided into three parts: 10% of the lysed samples were used as the control group and designated as "input"; 80% of the lysed samples were used for the immunoprecipitation reaction with anti-YTHDF2 antibodies and designated as "IP"; 10% of the lysed samples were incubated with rabbit-derived IgG (Cell Signaling Technology) as the negative control group and designated as "IgG".

4. The corresponding antibodies for each group were incubated separately and left to incubate overnight at 4°C. Pre-cleaned magnetic beads were then added, and the mixture was further incubated at 4°C for 2–4 hours to facilitate binding of the antibodies to the magnetic beads. The magnetic beads were subsequently washed 3–5 times with RIP washing buffer to remove non-specifically bound RNA. Elution buffer was added to the magnetic beads to elute the bound RNA. The eluted RNA was then purified to remove residual proteins and other impurities.

5. The single-stranded RNA sequencing library was constructed using the Illumina KC Digital™ Stranded mRNA Library Prep Kit (item number DR08502). This kit contains a unique molecular identifier (UMI) to label pre-amplified cDNA molecules, which is used to eliminate PCR and sequencing duplication bias.

6. The library products corresponding to 200-500 bps were enriched, quantified, and subjected to final sequencing using the PE150 model on the DNBSEQ-T7 sequencer (MGI Technology).

Data Processing:

1. Trimomatic (version 0.36) was employed to filter the raw data, retaining only high-quality sequences. Further data processing was then conducted using internal scripts.

2. The processed data were subsequently used to analyze protein binding sites with STAR software (version 2.5.3a), utilizing default parameters. Additional analysis and processing were performed using RSeQC (version 2.6), exomePeak (version 3.8), bedtools (version 2.25.0), deeptools (version 2.4.1), and Homer (version 4.10).

MeRIP-seq experimental steps and materials

MeRIP Experiment and Library Preparation:

1. Cell samples were prepared, and total RNA was extracted for quality testing, which included detecting RNA concentration and total amount, assessing purity, and evaluating completeness.

2. Total RNA was purified using the RNA Clean & Concentrator-5 kit. The RNA product was fragmented in the presence of metal ions, yielding RNA fragments with a length range of 60–200 bp. Half of the fragmented RNA was retained as input RNA, while the remaining RNA was used for the subsequent antibody immunoprecipitation step.

3. The M6A antibody was immunoprecipitated by pre-mixing Dynabeads Protein A magnetic beads with the antibody, followed by incubation at 4°C for 2 hours to form a complex of magnetic beads and antibody. Fragmented RNA was added to the magnetic bead-antibody solution and incubated at 4°C for 2 hours to allow RNA fragments with m6A methylation modification to bind to the antibody. The magnetic beads were separated from the solution using a magnetic rack, the solution was removed, and the magnetic beads were resuspended in a wash buffer and washed thoroughly 3–5 times. Eluent was added to the magnetic beads to elute RNA fragments containing the m6A modification, which were labeled as "IP".

4. The SMARTer® Stranded Total RNA Seq Kit v2 (Takara, JPN) was used for subsequent library preparation, following the instructions provided in the kit manual.

MeRIP sequencing

The library was sequenced using the Illumina high-throughput sequencing platform with a 2 × 150 bp paired-end sequencing strategy.

RIP-qPCR

Sample pre-processing was performed in the same manner as for RIP-seq. Specifically, the purified RNA was reverse transcribed into cDNA, which was then subjected to RT-qPCR analysis.
