# Supplementary material for: YTHDF2 enhances proliferation and metastasis of nasopharyngeal carcinoma by mediating m6A modification in destabilizing FOXO1 mRNA
Source: Cancer Biol Ther. 2025 Dec 10;26(1):2582349. doi: 10.1080/15384047.2025.2582349 (PMC12698064; doi:10.1080/15384047.2025.2582349)
Supplement: Supplementary Material — 3. [file KCBT_A_2582349_SM1422.docx]

Supplementary Material 2

1. Cell line and tissue RNA extraction

In this study, total RNA was extracted from tissues and cells using the RNA Rapid Tissue/Cell Kit (TIANGEN, DP451). The extraction procedure was conducted in strict accordance with the manufacturer's instructions. The Nanodrop 2000 was employed for the determination of RNA concentration and purity. The ratio of OD₂₆₀ to OD₂₈₀ (OD₂₆₀/OD₂₈₀) was used to reflect RNA quality.

2. RNA reverse transcription

RNA reverse transcription was performed using a two-step reverse transcription kit, which included DNase I, RNase-free (ThermoFisher, EN0529) and RevertAid MM (ThermoFisher, M1631). The reaction system was configured strictly according to the manufacturer's instructions. The specific reaction system and procedure are detailed in Table 1.

Table 1 System for RNA reverse transcription

| Component | Volume (10μL) |
| --- | --- |
| Total RNA | 1μg |
| DNase I | 1ul |
| Mgcl_2_ | 1ul |
| RNase-free Water | variable |

The eight connected tubes containing the prepared reaction system were placed into the PCR amplification instrument. The experimental procedure was as follows: incubation at 37℃ for 30 minutes. Subsequently, the eight tubes were removed, and 1 μL of EDTA was added to each reaction system, followed by incubation at 65℃ for 10 minutes.

Table 1 System for RNA reverse transcription (continue)

| Component | Volume (20μL) |
| --- | --- |
| The above reaction products | 9μL |
| 20🞨Enzyme Mix | 1μL |
| 2🞨RT Buffer Mix | 10μL |

Next, the eight connected tubes containing the prepared reaction system were placed into the PCR amplification instrument. The experimental procedure was as follows: incubation at 42℃ for 60 minutes, followed by 70℃ for 5 minutes. The configuration of the reverse transcription reaction system was performed on ice to maintain optimal conditions. The resulting cDNA was then stored in a -80℃ freezer for long-term preservation.

3. RT-qPCR

The PCR primers used in this study were shown in Table 2

Table 2 RT-qPCR primer table

| Primers | Sequences |
| --- | --- |
| FOXO1 | Forward 5’-TCGTCATAATCTGTCCCTACACA-3’ |
|  | Reverse 5’-CGGCTTCGGCTCTTAGCAAA-3’ |
| GAPDH | Forward 5’-GCTCAGACACCATGGGGAAG-3’ |
|  | Reverse 5’-TGTAGTTGAGGTCAATGAAGGGG-3’ |
| YTHDF2 | Forward 5’- AGCCCCACTTCCTACCAGATG-3’ |
|  | Reverse 5’- TGAGAACTGTTATTTCCCCATGC-3’ |
