# Supplementary material for: YTHDF2 enhances proliferation and metastasis of nasopharyngeal carcinoma by mediating m6A modification in destabilizing FOXO1 mRNA
Source: Cancer Biol Ther. 2025 Dec 10;26(1):2582349. doi: 10.1080/15384047.2025.2582349 (PMC12698064; doi:10.1080/15384047.2025.2582349)
Supplement: Supplementary Material — 2. [file KCBT_A_2582349_SM1423.docx]

Supplementary Material 3

Cell lines with overexpression of target genes:

Using liposome transfection technology, the plasmid carrying the target gene/blank (vector pCMV6-Entry) was transfected into NPC cell lines.

(1) The consumables and reagents required for subsequent experiments are placed on an ultraclean workbench and exposed to ultraviolet radiation for at least 30 minutes.

(2) Observe the growth status of the cells, digest the NPC cell line 5-8F in logarithmic growth phase, and evenly inoculate it into a six well plate. Transfection can only be performed when the cells have grown to cover 70% to 80% of the surface area of the culture well.

(3) Configure transfection system (with one well as one transfection reaction): Add 2.5 μg FOXO1 plasmid and 5 μL P3000 solution to 125 μL Opti-MEM to obtain a diluted plasmid solution, and add 3.75 μL Lipofectamine 3000 to 125 μL Opti-MEM to obtain a diluted Lipofectamine 3000 solution. Add the diluted plasmid solution to the diluted Lipofectamine 3000 solution, mix well, and let it stand at room temperature for 15 minutes.

(4) Remove the culture medium from the six well plate, wash the cells once with 1 × PBS buffer, add complete culture medium without antibiotics, slowly add the prepared transfection system mixture to the six well plate, make the total volume of liquid in each well 2 mL, gently shake the six well plate, and place it in the incubator for cultivation.

(5) Transfect for 24 hours, remove the culture medium, wash the cells with 1 × PBS buffer, and conduct subsequent experiments.

(6) qRT-PCR was used to detect overexpression effects.

Cell lines with knockdown of target genes:

Using siRNA interference technology, artificially synthesized siRNA with target gene/blank knockout function is introduced into cell lines.

(1) The consumables and reagents required for subsequent experiments are placed on an ultraclean workbench and exposed to ultraviolet radiation for at least 30 minutes.

(2) Observe the growth status of the cells, digest the NPC cell lines in logarithmic growth phase, and evenly inoculate them into a six well plate. Transfection can only be carried out when the cells have grown to cover 70% to 80% of the surface area of the culture well (the cell density can be adjusted according to the cell state during transfection).

(3) Dilute siRNA with serum reducing medium Opti-MEM, add 1.5 μL siRNA to 150 μL Opti-MEM at a concentration of 20 μM, and mix gently.

(4) Dilute the transfection reagent lipoRNAiMAX with serum reducing medium Opti-MEM, add 150 μL of Opti-MEM to 9 μL of transfection reagent, and gently mix well

(5) Mix the diluted siRNA with transfection reagent lipoRNAiMAX, gently blow it evenly, and let it stand at room temperature for 5 minutes.

(6) Discard the culture medium from the six well plate, wash the cells once with 1 × PBS buffer, add complete culture medium without antibiotics, slowly add the prepared transfection system mixture to the six well plate, make the total volume of liquid in each well 2 mL, gently shake the six well plate, and place it in the incubator for cultivation.

(7) Transfect for 24 hours, remove the culture medium, wash the cells with 1 × PBS buffer, and conduct subsequent experiments.

(8) qRT-PCR was used to detect the knockdown effect.
