# Supplementary material for: YTHDF2 enhances proliferation and metastasis of nasopharyngeal carcinoma by mediating m6A modification in destabilizing FOXO1 mRNA
Source: Cancer Biol Ther. 2025 Dec 10;26(1):2582349. doi: 10.1080/15384047.2025.2582349 (PMC12698064; doi:10.1080/15384047.2025.2582349)
Supplement: Supplementary Material — 1. [file KCBT_A_2582349_SM1424.docx]

Supplementary Material 1

Patient information (primary fresh NPC tissue, M0)

| gender | age | T stage | N stage | Clinical Stage |
| --- | --- | --- | --- | --- |
| male | 46 |  |  |  |
| male | 50 | 4 | 2 | ⅣA |
| male | 59 |  |  |  |
| male | 40 | 3 | 3 | ⅣA |
| male | 44 | 4 | 2 | ⅣA |
| female | 49 |  |  |  |
| male | 75 |  |  |  |
| male | 58 | 2 | 1 | Ⅱ |
| male | 58 | 3 | 1 | Ⅲ |
| male | 47 | 4 | 2 | ⅣA |
| male | 33 |  |  |  |
| female | 51 | 3 | 3 | ⅣA |
| female | 59 | 2 | 2 | Ⅲ |
| female | 59 | 4 | 3 |  |
| female | 49 |  |  |  |
| male | 35 |  |  |  |
| female | 17 | 3 | 3 | ⅣA |

Patient information (rhinitis tissue)

| gender | age | clinical diagnosis |
| --- | --- | --- |
| female | 41 | chronic rhinosinusitis |
| male | 20 | chronic rhinosinusitis |
| male | 37 | chronic rhinosinusitis |
| female | 48 | chronic rhinitis |
| male | 39 | Nasopharyngeal mass |
| female | 58 | rhinosinusitis |
| female | 59 | Right nasal bleeding |
| male | 4 | bronchitis |
| female | 33 | rhinosinusitis |
| male | 31 | cyst of paranasal sinuses |
| male | 22 | rhinosinusitis |
| male | 71 | rhinosinusitis |
| male | 56 | nasal polyp |
| female | 48 | rhinosinusitis |
| female | 27 | rhinosinusitis |

Patient information (paraffin-embedded rhinitis tissue)

| gender | age | clinical diagnosis |
| --- | --- | --- |
| male | 59 | chronic rhinosinusitis |
| female | 59 | rhinosinusitis |
| male | 67 | rhinosinusitis |
| male | 26 | rhinosinusitis |
| male | 61 | rhinosinusitis |
| male | 57 | rhinosinusitis |
| female | 68 | rhinosinusitis |
| male | 44 | rhinosinusitis |
| female | 53 | rhinosinusitis |
| male | 23 | rhinosinusitis |
| male | 19 | rhinosinusitis |
| male | 18 | chronic rhinosinusitis |
| male | 44 | chronic rhinosinusitis |
| male | 41 | rhinosinusitis |
| male | 32 | Deviated Nasal Septum |
| female | 76 | Deviated Nasal Septum |
| female | 13 | rhinosinusitis |
| male | 51 | rhinosinusitis |
| female | 51 | chronic rhinosinusitis |
| female | 72 | rhinosinusitis |
| male | 18 | rhinosinusitis |
| male | 14 | rhinosinusitis |
| female | 18 | rhinosinusitis |
| male | 40 | rhinosinusitis |
| male | 22 | rhinosinusitis |
| male | 55 | rhinosinusitis |

Patient information (paraffin-embedded NPC tissue)

| gender | age | T stage | N stage | M stage | Clinical Stage |
| --- | --- | --- | --- | --- | --- |
| male | 54 | 3 | 3 | 0 | ⅣA |
| male | 35 | 3 | 3 | 0 | ⅣA |
| male | 30 | 2 | 3 | 0 | ⅣA |
| male | 41 | 3 | 3 | 0 | ⅣA |
| female | 66 | 3 | 3 | 0 | ⅣA |
| male | 63 | 4 | 3 | 0 | ⅣA |
| male | 66 | 4 | 1 | 1 | ⅣB |
| male | 65 | 2 | 3 | 0 | ⅣA |
| male | 46 | 2 | 3 | 0 | ⅣA |
| male | 36 | 4 | 3 | 1 | ⅣB |
| male | 39 | 4 | 3 | 0 | ⅣA |
| male | 62 | 2 | 3 | 0 | ⅣA |
| male | 68 | 4 | 1 | 0 | ⅣA |
| female | 44 | 3 | 3 | 0 | ⅣA |
| male | 65 | 4 | 3 | 0 | ⅣA |
| male | 53 | 4 | 2 | 1 | ⅣB |
| male | 56 | 3 | 3 | 0 | ⅣA |
| male | 36 | 4 | 3 | 0 | ⅣA |
| male | 57 | 4 | 2 | 0 | ⅣA |
| male | 55 | 3 | 3 | 1 | IVB |
| male | 13 | 4 | 2 | 0 | ⅣA |
| female | 66 | 4 | 3 | 1 | IVB |
| female | 57 | 3 | 3 | 1 | IVb |
| male | 55 | 4 | 3 | 1 | ⅣB |
| female | 38 | 3 | 3 | 1 | ⅣB |
| female | 27 | 2 | 2 | 0 | Ⅲ |
| male | 60 | 2 | 1 | 0 | Ⅱ |
| male | 38 | 3 | 3 | 0 | Ⅳa |
| male | 55 | 3 | 2 | 0 | Ⅲ |
| female | 60 | 2 | 3 | 0 | Ⅳa |
| male | 51 | 3 | 2 | 0 | Ⅲ |
| male | 34 | 4 | 2 | 0 | Ⅳa |
| male | 46 | 4 | 2 | 0 | Ⅳa |
| male | 69 | 2 | 3 | 0 | Ⅳa |
| male | 59 | 3 | 3 | 0 | Ⅳa |
| male | 28 | 4 | 3 | 1 | Ⅳb |
| male | 66 | 1 | 3 | 0 | Ⅳa |
| male | 47 | 4 | 2 | 0 | Ⅳa |
| male | 51 | 4 | 1 | 0 | Ⅳa |
| male | 25 | 4 | 3 | 0 | Ⅳa |
| female | 47 | 3 | 2 | 0 | Ⅲ |
| male | 61 | 4 | 3 | 1 | Ⅳb |
| male | 37 | 4 | 1 | 0 | Ⅳa |
| male | 54 | 4 | 2 | 0 | Ⅳa |
| male | 46 | 4 | 3 | 0 | Ⅳa |
