# Supplementary material for: YTHDF2 enhances proliferation and metastasis of nasopharyngeal carcinoma by mediating m6A modification in destabilizing FOXO1 mRNA
Source: Cancer Biol Ther. 2025 Dec 10;26(1):2582349. doi: 10.1080/15384047.2025.2582349 (PMC12698064; doi:10.1080/15384047.2025.2582349)
Supplement: Supplementary Material — Figure Legends. [file KCBT_A_2582349_SM1421.docx]

Figure 1. Analysis of the expression of YTHDF2 and its impact on the progression of NPC. (A) Comparison of the transcription levels of YTHDF2 in the combined GSE12452, GSE180272, GSE53819, GSE61218, and GSE64634 datasets. (B) Comparison of the expression level of YTHDF2 between malignant and nonmalignant cells in single-cell NPC dataset GSE150430. (C) ROC curve analysis of YTHDF2 in diagnosing NPC. (D) Kaplan–Meier survival curve for YTHDF2 expression of NPC in GSE102349.

Figure 2. The expression of FOXO1 in the transcription and translation of NPC cells and tissues.

(A) Comparison of the transcription level of YTHDF2 in NPC cell lines with NP69. (B) Comparison of the transcription level of YTHDF2 in NPC tissues with Rhinitis.

(A: n = 3 biological replicates, one-way ANOVA, B: t-test). (C-D) Comparison of the protein level of YTHDF2 in NPC cell lines with NP69. (E-F) Comparison of the protein level of YTHDF2 in NPC tissues with Rhinitis. (D: n = 3 biological replicates, one-way ANOVA, F: t-test). * P < 0.05; ** P < 0.01; *** P < 0.001; **** P < 0.0001; ns: not significant.

Figure 3. *In vitro* functional experiments of YTHDF2 knockdown in NPC cells (n = 3 biological replicates, student's t-test). (A-C) YTHDF2 knockdown inhibited the proliferation in 5-8F, CNE1 and HONE1cells. (D-F) YTHDF2 knockdown reduced the migration in 5-8F, CNE1 and HONE1 cells. YTHDF2 knockdown reduced the invasive capabilities of 5-8F, CNE1 and HONE1 cells. * P < 0.05; ** P < 0.01; *** P < 0.001; **** P < 0.0001.

Figure 4. *In vitro* functional experiments of YTHDF2 overexpression in NPC cells (n = 3 biological replicates, student's t-test). (A-C) YTHDF2-transfected 5-8F, CNE1, and HONE1 cells exhibited accelerated proliferation. (D-F) Enhanced migratory capacity was observed in YTHDF2-overexpressing cells. (G-H) Matrigel invasion assays revealed significantly increased invasiveness following YTHDF2 overexpression. * P < 0.05; ** P < 0.01; *** P < 0.001; **** P < 0.0001.

Figure 5. Correlation between YTHDF2 and FOXO1 expression (n = 3 biological replicates, C-D: t-test, E-F: one-way ANOVA). (A) GSEA in GSE102349 revealed enrichment pathways in the YTHDF2-activated group. (B) Correlation analysis between YTHDF2 and FOXO1 expression in GSE102349. Analysis of FOXO1 expression in 5-8F and CNE1 cells following YTHDF2 knockdown (C) or overexpression (D). Comparison of FOXO1 expression in NPC cell lines 5-8F (E) and CNE1 (F) which were treated with the m6A demethylase inhibitor 3-DAA for 48 h.* P < 0.05; ** P < 0.01; *** P < 0.001; ns: not significant.

Figure 6. YTHDF2 participates in m6A-mediated regulation of FOXO1. (A) Distribution of m6A modification sites of FOXO1 mRNA in 5-8F. (B) Volcano plot of MeRIP-seq analysis for YTHDF2. (C) RIP-qPCR exhibited the relative expression of FOXO1. (D) YFHDF2 binding interval in FOXO1. (E) M6A modification site on the 3'UTR of FOXO1.
